# A Semi-Automated Workflow for Brain Slice Histology Alignment, Registration, and Cell Quantification (SHARCQ)

**DOI:** 10.1523/ENEURO.0483-21.2022

**Published:** 2022-04-19

**Authors:** Kristoffer Lauridsen, Annie Ly, Emily D. Prévost, Connor McNulty, Dillon J. McGovern, Jian Wei Tay, Joseph Dragavon, David H. Root

**Affiliations:** 1 Department of Psychology and Neuroscience, University of Colorado, Boulder, CO 80301; 2 BioFrontiers Institute, University of Colorado, Boulder, CO 80303

**Keywords:** atlas registration, brain mapping, cell-type

## Abstract

Tools for refined cell-specific targeting have significantly contributed to understanding the characteristics and dynamics of distinct cellular populations by brain region. While advanced cell-labeling methods have accelerated the field of neuroscience, specifically in brain mapping, there remains a need to quantify and analyze the data. Here, by modifying a toolkit that localizes electrodes to brain regions (SHARP-Track; Slice Histology Alignment, Registration, and Probe-Track analysis), we introduce a post-imaging analysis tool to map histological images to established mouse brain atlases called SHARCQ (Slice Histology Alignment, Registration, and Cell Quantification). The program requires MATLAB, histological images, and either a manual or automatic cell count of the unprocessed images. SHARCQ simplifies the post-imaging analysis pipeline with a step-by-step GUI. We demonstrate that SHARCQ can be applied for a variety of mouse brain images, regardless of histology technique. In addition, SHARCQ rectifies discrepancies in mouse brain region borders between atlases by allowing the user to select between the Allen Brain Atlas or the digitized and modified Franklin–Paxinos Atlas for quantifying cell counts by region. SHARCQ produces quantitative and qualitative data, including counts of brain-wide region populations and a 3D model of registered cells within the atlas space. In summary, SHARCQ was designed as a neuroscience post-imaging analysis tool for cell-to-brain registration and quantification with a simple, accessible interface. All code is open-source and available for download (https://github.com/wildrootlab/SHARCQ).

## Visual Abstract

**Figure:**
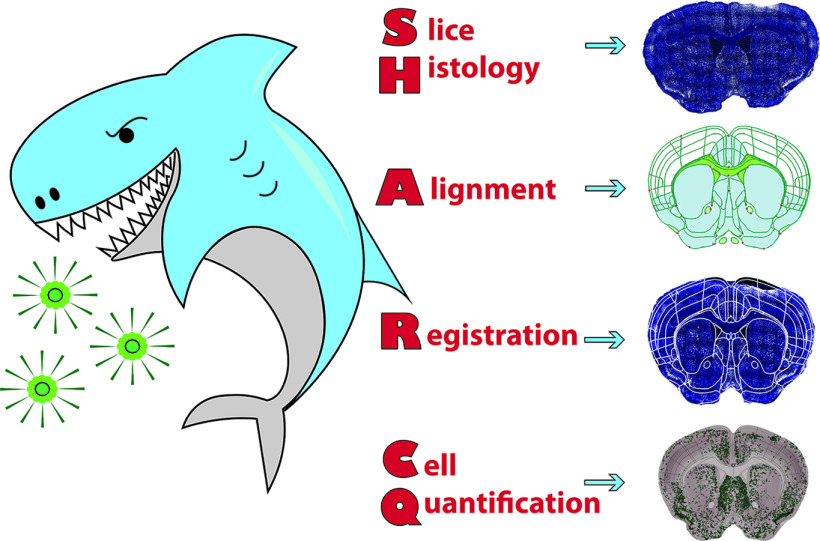

## Significance Statement

Advancements in genetically distinct cell-labeling have begun to outpace the analysis required to quantify it. Particularly for whole-brain mapping, robust and automated methods for quick post-imaging analysis have become increasingly necessary to process large amounts of data contained in images. To address the need for processing high-throughput imaging data, we developed a tool to count fluorescent cells by brain region using the digitized Allen Brain Atlas and the modified Franklin–Paxinos Atlas. This tool is called SHARCQ (Slice Histology Alignment, Registration, and Cell Quantification).

## Introduction

**Figure 1.**
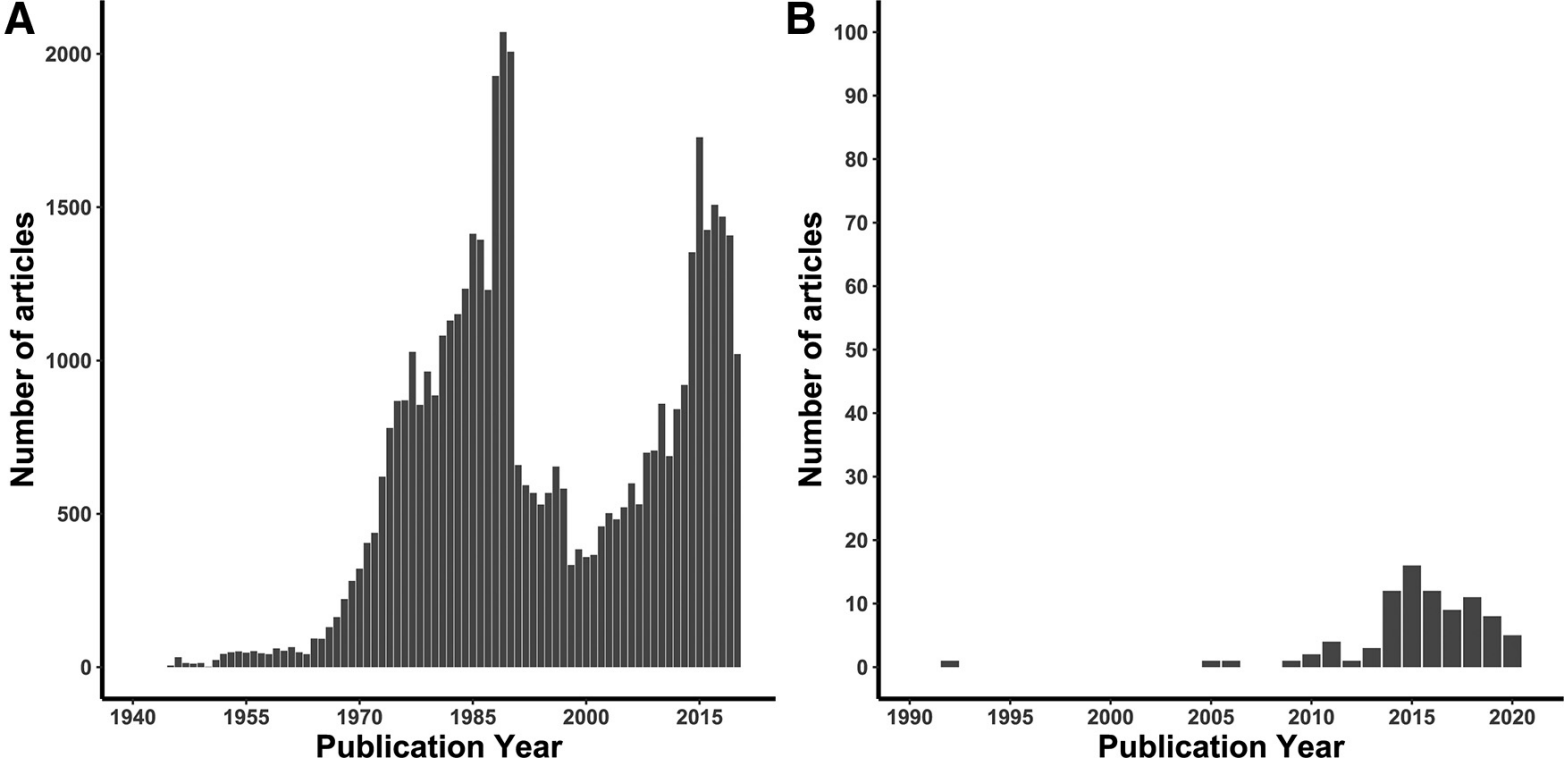
Trend in histology-related keywords in neuroscience-based peer-reviewed article publications from the Scopus database. ***A***, Articles from 1912 to 2020 that contained “histology” as a keyword. ***B***, Articles from 1992 to 2020 containing the keywords “automated, brain atlas, histology.”

A major neuroscientific goal is to delineate the cytoarchitectural organization of the brain. The discovery of population-like cell body clusters prompted the demarcation of distinct brain territories, which in turn developed into whole-brain atlases with standardized nomenclature across multiple species, including rodents (Lein et al., 2007; Franklin and Paxinos, 2013; Paxinos and Watson, 2014). These brain atlases have their origins in morphological and some immunohistochemical preparations. However, transcriptional and genetic assays indicate far more cell-types across the brain than have been previously appreciated (Welch et al., 2019; Yao et al., 2021). Current histological processes involve targeted protein or gene expression and light microscopy to generate qualitative data in the form of brain section images that fluorescently or chromogenically label neurons of interest. The modern neuroscientist exists in a scientific renaissance in which there are neuroanatomical methods that have become ever more complex and refined for interrogating the genetic and circuit identity of neurons (Saleeba et al., 2019). Yet there are still few options for robust, reproducible, and easily accessible post-imaging analysis to map histology to brain atlases. This can be depicted by the trend of publications with the keyword “histology” versus publications that include “automated, brain atlas, and histology” as keywords (Fig. 1). As the techniques for neuro-histology have improved and their scale of use has increased, development in the methods used for registering and quantifying histology data within existing, standardized atlas spaces has become necessary. While automated cell counting options exist (Brey et al., 2003; Kopec et al., 2011; Schüffler et al., 2013; Morriss et al., 2020; Courtney et al., 2021; Paglia et al., 2021; Sekar et al., 2021), few take into consideration the unbiased approach of whole-brain mapping, thus requiring a pipeline for cell counting that includes registering histology to border-defined structures in the whole brain. In the context of whole-brain imaging and mapping, where the volume of data is exceedingly large, the need for these processes to become automated has become apparent.

Advances in digital-based atlases (Hjornevik et al., 2007; Lein et al., 2007) set the stage for the development of tools for registering histology to atlas reference spaces (Kopec et al., 2011; Pallast et al., 2019). Atlas registration along with the quantification of brain region populations for labeled cells are critical steps for accurate post-imaging analysis. Atlas registration involves a warping mechanism to align histology sections onto an idealized atlas (Kopec et al., 2011; Shamash et al., 2018; Pallast et al., 2019; Bourgeois et al., 2021; Claudi et al., 2021). Deformations, angled images from non-zero anterior-posterior or medial-lateral tilt, and other imperfections in the slice image can complicate the warping process that is necessary to map sections to the atlas space. Therefore, accuracy in atlas registration is essential.

The SHARP-Track toolkit (Slice Histology Alignment, Registration, and Probe-Track analysis) was developed as a method for user-identification of landmark points between a section image and the mouse brain atlas to accurately register histology data containing electrode tracks, thus accommodating tissue imperfections (Shamash et al., 2018). The SHARP-Track toolkit allows intuitive interaction with the Allen CCF Mouse Brain Atlas (Wang et al., 2020) and can incorporate images collected on various angles. Here, we introduce a modified SHARP-Track that is a semi-automated pipeline of slice to mouse brain atlas registration and cell counting of fluorescently labeled neurons called SHARCQ (Slice Histology Alignment, Registration, and Cell Quantification).

## Materials and Methods

### Whole-brain fluorescent labeling of neurons

Male and female VGluT2-IRES::Cre mice (10–16 weeks) were anesthetized with isoflurane (1–3%) and injected in the ventral tegmental area (VTA) with adeno-associated viral vectors (AAVs) encoding Cre-dependent avian tumor virus receptor A (TVA) tethered to mCherry and Cre-dependent optimized glycoprotein (Kim et al., 2016; AP: −3.2, ML: 0, DV: −4.3). Three weeks later, mice were anesthetized with isoflurane (1–3%) and injected in VTA with EnvA G-deleted rabies GFP (Salk Institute). One week following rabies injection, mice were anesthetized with isoflurane and transcardially perfused with phosphate buffer (0.1 m) followed by 4% paraformaldehyde. Brains were left in paraformaldehyde for 2 h and transferred to 18% sucrose in phosphate buffer overnight at 4°C. Brains were frozen in dry ice, sectioned (30 μm) in the coronal plane, mounted to gelatin-coated slides, and cover-slipped with Prolong Diamond containing DAPI, while minimizing tissue folding and the appearance of bubbles. DAPI was used as the cell marker for histology, and it is highly advised for the purposes of SHARCQ to use any cell marker as a background channel to easily discern the edges and landmarks of brain sections. Cover-slipped slides were weighed down by a wellplate for 3 days to promote a flat imaging plane across the entire slide. For some sections before cover-slipping, guinea pig anti-c-Fos (Sysy, 1:1000) or mouse anti-tyrosine hydroxylase (TH; Millipore, 1:500) were washed overnight at 4°C and secondaries were applied for 2 h at room temperature (anti-guinea pig Alexa Fluor 647, anti-mouse DyLight405, or anti-mouse Alexa Fluor 488, The Jackson Laboratory, 1:200).

### Image acquisition

Images were collected with confocal microscopy using a PerkinElmer Opera Phenix High-Content Screening System using a 20× 0.8 NA objective. All images were obtained as 16-bit multichannel images. Individual fluorophore channels were stitched together on a composite .tiff image using a custom-written MATLAB script (StitchLargeImage.m) available for download at https://www.root-lab.org/.

### Code accessibility: installation and dependencies

The SHARCQ program uses functionality from the MATLAB SHARP-Track toolkit (https://github.com/cortex-lab/allenCCF) with additional developments and a graphical user interface (GUI) that registers the Allen Mouse Brain Atlas onto brain slice images and quantifiable cell locations. The SHARCQ program can be accessed through the SHARCQ GitHub repository, which contains a full outline of installation, detailed requirements, and methods of use (https://github.com/wildrootlab/SHARCQ). The 2020b or later installation of MATLAB is required, along with the Image Processing toolbox. The reference brain atlas that was used to overlay brain regions over slice images was the Allen Institute’s 10-μm voxel 2017 version from the Allen Mouse Brain Common Coordinate Framework version 3 (Wang et al., 2020). Cell quantification by brain region can be completed with either the Allen Mouse Brain Atlas (Wang et al., 2020) or the modified Franklin–Paxinos atlas (Chon et al., 2019).

### Manual cell counting

The original 16-bit acquired images were opened in FIJI for experimenter-blinded counts of GFP-labeled cell bodies. If the images were in the incorrect anatomic orientation, they were rotated to the correct coronal orientation (dorsal side is “up” and ventral side is “down”) and saved before starting manual cell counting. To avoid error, the X,Y coordinate file must be generated from an image of the same orientation, size, and aspect ratio. The “Multi-Point” tool was used to label cell body locations over the image. The X,Y coordinates of counted cells were exported as a .csv file using the “measure” function. The same acquired images were also counted in Photoshop to assess the accuracy of our pipeline in counting cells from different software for quantification. The X,Y coordinates of counted cells were extracted using a custom-written JavaScript script (get_xy_photoshop.js), which is included in the SHARCQ GitHub repository.

### Image pre-processing

The three-channel stitched histology .tif images and X,Y coordinate .csv or .txt files from cell counting were manually moved into the “Images” and “ROI Coordinates” subfolders, respectively, of the SHARCQ-master folder. Through the first step of the SHARCQ GUI, the contrast was manually adjusted using the imcontrast MATLAB function to increase visibility of landmarks. A region of interest (ROI) file for each image was automatically created by SHARCQ and stored in a newly generated “Processed” folder within the “Images” subfolder. The ROI file represents the counted cell locations in a binary matrix that equals the image size and aspect ratio. Both the image and ROI matrices were downsampled to match the resolution of the reference atlas (10 μm/pixel). After all files were processed in this way, a new window was prompted. The user must click through and review all images using the arrow keys. This step filled the borders of the images and ROI matrices to 800 × 1140 pixels to match the aspect ratio of the Allen atlas for image-atlas registration.

### Image-atlas registration

Using both the “Slice Viewer” and “Atlas Viewer” figures generated in the second step of the GUI, we registered slice images to reference slices in the Allen atlas by scrolling to the approximate bregma location in the Atlas Viewer window (Wang et al., 2020). Where applicable, we tilted the atlas image in the dorsoventral and mediolateral planes to match the slice image. Finding the approximate bregma location and tilt is at the discretion of the user, and it is advised that the user has moderate familiarity with neuroanatomy. It is recommended to use white matter tracts (i.e. anterior commissure, corpus callosum, medial lemniscus, etc.) as landmarks. After navigating to the approximate bregma location and tilt, at least 10 points of reference were clicked that matched between the Atlas Viewer and the Slice Viewer. SHARCQ later used these points of reference to transform and overlay the slice image over the atlas image, as well as transforming the ROI matrix with the cell locations.

### ROI-atlas registration

Registration of the slice image to a reference atlas slice in the second step of the GUI generates a geometric transformation file. In the third step, this geometric transformation was applied to the separate ROI layer via the Warp_ROI.m script located under the “Code” and then the “ROI” folder of SHARCQ-master. As a result, the ROI matrix containing cell locations is geometrically warped onto the atlas image in an identical way to the slice image.

### Image-ROI-atlas composition and quantification by brain region

In the fourth step of the GUI, the warped image-to-atlas and ROI-to-atlas files were compiled in the Analyze_ROI.m script to create a composite image with three overlays: the downsampled image, the counted cell locations, and the atlas slice borders. The Analyze_ROI.m script is stored under the “Code” then “ROI” folder of SHARCQ-master. At this step, the user can add, move, or delete the fully interactive ROI points to account for any insurmountable difficulties in registration. The output is one .txt file per slice image containing a list of each counted cell’s brain region location and one slice image-atlas-ROI composite .tif file.

### ROI-3D brain registration

In the fifth step of the GUI, the ROIs from all brain slice sections that were analyzed within the “Images” folder were compiled into one 3D rendering of the mouse brain. For 3D registration, all SHARCQ output .csv files generated in the fourth step should be contained in the following folder path: Images→Processed→ROIs→Brain_Points. 3D registration was accomplished with the Plot_Wire_Frame_Brain.m script under “Code” and then the “ROI” folder of SHARCQ-master.

### Brain-wide ROI quantification by brain region

In the sixth and final step of the GUI, all SHARCQ output .csv files generated in the fourth step were compiled into one .csv file per brain. The final result is an automatic tally of the number of counted cells by brain region across the entire analyzed brain.

## Results

### Flowchart of the initial set-up and organization of SHARCQ folders required for operation

**Figure 2.**
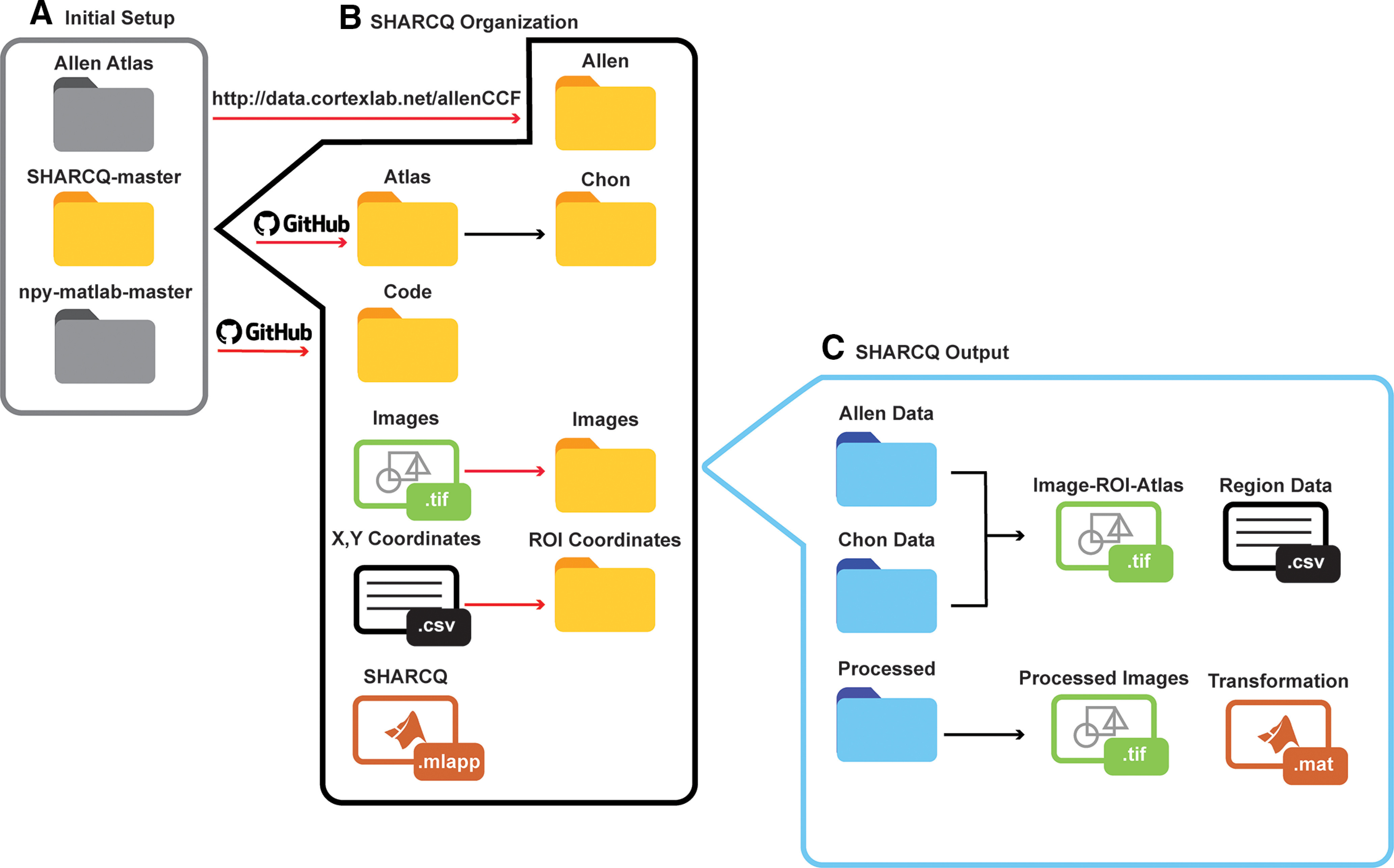
Setup and SHARCQ GitHub repository file organization. ***A***, The pipeline for SHARCQ requires downloading the SHARCQ dependencies, which include the SHARCQ GitHub repository, the Allen Atlas from http://data.cortexlab.net/allenCCF, and the npy-matlab GitHub repository. Red arrows denote required user action. ***B***, There are several folders organized by content within the SHARCQ-master folder. Before opening the SHARCQ.mlapp, the user must input images and the corresponding X,Y coordinate files in the appropriate folders in SHARCQ-master. ***C***, SHARCQ generates new folders and files within the Images folder. The overlayed image-ROI-Atlas.tif file and cell counts by region data.csv file are both stored in the corresponding Allen Data or Chon Data folder depending on the selected atlas. A new folder called “Processed” contains images and transformation data that were used during the image-ROI-atlas registration steps of SHARCQ.

Initial configuration of SHARCQ requires user-input at discrete steps. First, the user must download the SHARCQ-master repository from GitHub, the npy-matlab-master repository from GitHub, as well as the Allen Atlas data (Fig. 2*A*). Once downloaded, the Allen Atlas content should be stored in the “Allen” folder within the “Atlas” folder of SHARCQ-master. Because of the data file size of the Allen Atlas, our pipeline requires the user to separately download the Allen Atlas. The npy-matlab-master folder, which reads .npy files into MATLAB and is required for use of the digital Allen Atlas, should be stored in the “Code” folder of SHARCQ-master.

The SHARCQ-master folder contains the SHARCQ.mlapp application file to execute the program within MATLAB and four folders that are organized based on content: Atlas, Code, Images, and ROI Coordinates (Fig. 2*B*). The “Atlas” folder is further subdivided by the “Allen” folder and the “Chon” folder. The “Chon” folder contains all content necessary for the modified Franklin–Paxinos Atlas-related analysis and does not necessitate user modification. The “Code” folder should contain the following folders for full operation: Browsing Functions, Cell Counting Code, Histology Functions, npy-matlab-master, ROI, and SHARP-Track. For the inexperienced programmer, there is no need to directly open or manipulate any of the scripts within the “Code” folder, except to add the downloaded npy-matlab-master folder. All scripts within the “Code” folder contribute to the functionality of the SHARCQ MATLAB app and require no modifications by the user.

The “Images” folder is where the user places .tif image files for processing. There should be corresponding X,Y coordinate .csv files or .txt files for each image, and these files should be stored in the “ROI Coordinates” folder. For practice, example images and corresponding X,Y coordinates have been provided in the GitHub repository. It is important to note that the file name for the image must be the same in the corresponding X,Y coordinate file. In addition, the X,Y coordinate file should be generated from an image in the correct anatomic orientation (dorsal side is “up” and ventral side is “down” for coronal section). Both the X,Y coordinate file and image should be saved and stored in the correct anatomic orientation in the corresponding folder. Clicking the SHARCQ.mlapp file in the SHARCQ-master folder will open the SHARCQ GUI in MATLAB. User interaction with SHARCQ will generate several new folders and files in the process (Fig. 2*C*). Within the “Images” folder, the image-ROI-atlas composite .tif file and the region data .csv file will be stored in the corresponding atlas data folder. A new folder within the “Images” folder called “Processed” contains the transformation data and images that were used during image-ROI-atlas registration.

### A simplified workflow for adapting multiple software for cell count quantification to atlas registration

**Figure 3.**
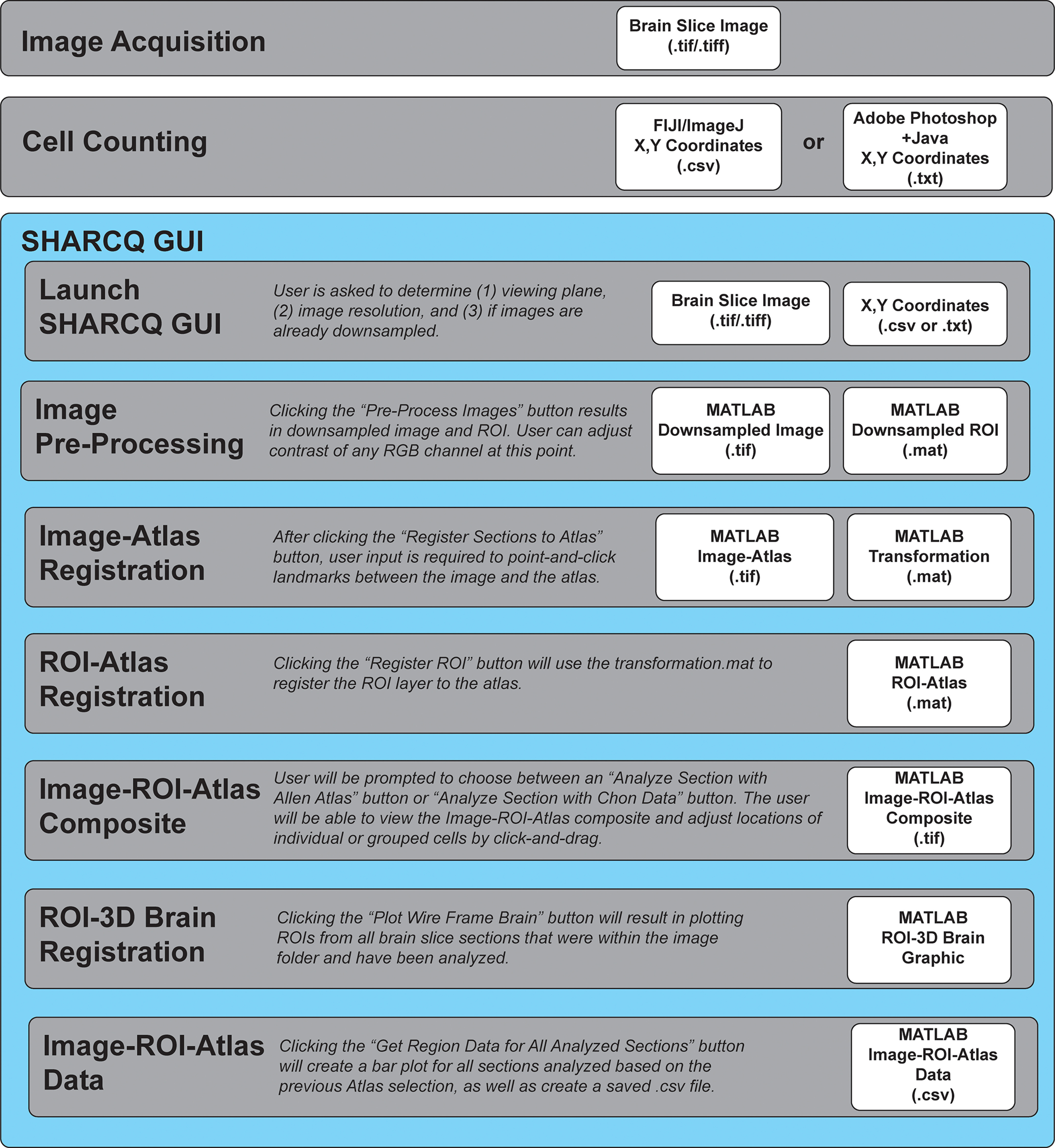
SHARCQ pipeline from image acquisition to cell counting and quantification. The brain slice image must be in a .tif/.tiff file format. If the image has multiple fluorescent channels, then the colors should be in RGB-TIF color formatting. There are a number of methods for cell counting, as long as the method generates a .csv or .txt file of X,Y coordinates of the counted cells within the dimensions of the image. Within the SHARCQ GUI, the user sets up the files that will become necessary for image-ROI-atlas registration. In the final three steps, the user has built-in options for visualization of data.

We created a workflow that utilizes secondary software for whole-brain slice imaging, cell quantification, and a MATLAB app that we designed for atlas registration (Fig. 3). The SHARCQ pipeline consists of three stages: image acquisition, cell counting, and the SHARCQ GUI. Understanding that there may be a variety of histology needs that contribute to different variables, such as number of channels, slice thickness, and image resolution, SHARCQ was designed so that the only image requirement at the acquisition stage is that it must be a .tif or .tiff file format. The next stage is cell counting. For the purposes of demonstrating the flexibility of the SHARCQ pipeline, we loaded images and manually counted cells using two different programs: FIJI and Adobe Photoshop. FIJI is an open-source image analysis software with manual cell counting capabilities in the GUI that also outputs an X,Y coordinate .csv file (Schindelin et al., 2012). Automatic cell counts can also be accomplished with the Analyze Particles command following thresholding and binarization. Adobe Photoshop is a commercial software that also has a “Count Tool” feature that can be purposed for manual cell counting. In order to export the X,Y coordinates as .txt files from the Adobe Photoshop cell counting feature, a Javascript file called “get_xy_photoshop.js” must be used. When these cell count files are moved into the correct folder, SHARCQ will automatically execute a separate MATLAB script called “importXY,” which will convert the .txt files to .csv files. Both scripts are stored in the “Cell Counting Code” folder within SHARCQ-master.

The bulk of user-input occurs within the SHARCQ GUI. After launching the SHARCQ.mlapp GUI, the user will be prompted to identify certain features of the images to be analyzed: viewing plane, image resolution, and downsampling. The viewing plane can either be coronal, sagittal, or transverse, but if sagittal, cell count quantification using the Chon Atlas cannot be accomplished at this time. Keeping in mind that the Allen Atlas is set at a resolution of 10 μm/pixel, downsampling an image means saving the image at a lower resolution to have it registered onto the Allen Atlas. The provided example images in the SHARCQ-master folder have a resolution of 0.5 μm/pixel. In the first step, the user is prompted to click the “Pre-Process Images” button. Doing so results in a downsampling of both the image and the created ROI matrix from the X,Y coordinates.

At the preprocessing image step, the user can adjust the contrast of any RGB channel to highlight certain landmarks that will make registration easier in the next step. In the prompted new window, the user must click through all images, if there are multiple channels, for review. This step saves the images and ROI matrices in the same aspect ratio as the Allen atlas, which is required for the next step of image to atlas registration. In the subsequent step, the user is prompted to click the “Register Sections to Atlas” button, which will allow for registration of the image to the appropriate coordinate within the mouse brain. This is a feature of the SHARP-Track toolkit, which has been integrated with SHARCQ with some modification, mainly adding numbers to landmarks to easily keep track of landmark positions between the “Atlas Viewer” window and “Slice Viewer” window. Instructions in the MATLAB command window are meant to direct the user on how to register the slice image to the atlas. In the “Atlas Viewer” window, the user must scroll to the approximate location in the brain that matches the slice in the “Slice Viewer” window. It is at the discretion of the user to determine the correct coordinate and tilt, if necessary. It is highly advised that at least 10 landmark points are clicked and matching between the “Atlas Viewer” and “Slice Viewer” windows. If the registration was successful, a “transform saved” message will appear in the MATLAB command window and an image-atlas composite .tif file as well as a transformation .mat file will be generated in the “Processed” folder.

The third step “Register ROI” requires no explicit user input, except clicking the button, which will register the ROI matrices using the same transformation .mat file that was applied to the image. If registration of the ROI was successful, then an “Image and ROI processing complete!” message will appear in the MATLAB command window. In the fourth step, the user may choose to quantify the cell counts by brain region according to the Allen Atlas or the Chon Atlas. In the MATLAB command window, the user will be prompted to select the original, unprocessed image file, which should be located in the “Images” folder. After doing so, a new window will appear, allowing the user to drag, add, or delete individual or grouped ROI points by drawing a polygon. When completed, the user is to press the “enter” key in the MATLAB command window. After doing so, SHARCQ will generate an image-ROI-atlas composite .tif file and a Region Data .csv file within the corresponding atlas folder in “Images.”

In the fifth step, the user can choose to plot the ROIs of all the images that have been processed thus far onto a 3D rendering of a mouse brain. No additional user input is required for this step. For the sixth and final step, the user can obtain the cumulative cell counts per brain region for all images that have been processed in a MATLAB generated plot. Otherwise, the user can replicate the same plot using the Region Data .csv file stored in “Images.”

### SHARCQ accurately registers both the histological image and ROI of counted cells to the atlas

**Figure 4.**
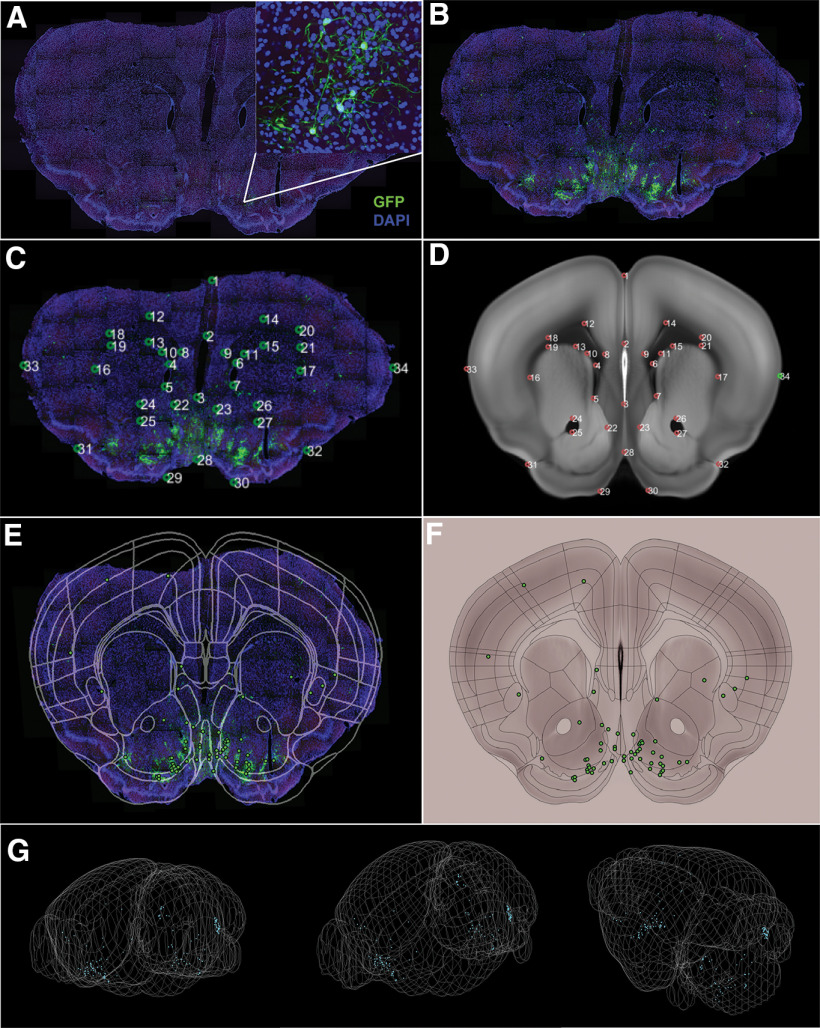
Demonstration of SHARCQ functionality in atlas and cell count ROI registration with imperfect histology. ***A***, Neuroanatomical tracing of neurons (green) projecting to VGluT2-expressing VTA neurons with whole cells in DAPI (blue) with the determined +1.35 AP coordinate. ***B***, Contrast adjustment of the image in the second step within SHARCQ GUI. ***C***, The “Slice Viewer” window with numbered matching landmarks between the (***D***) “Atlas Viewer” window. ***E***, Image-ROI-Allen Brain Atlas composite. ***F***, Image-ROI-Chon Atlas composite. ***G***, 3D whole-brain mapping of cell counts from all images that have been processed.

Here, for purposes of demonstration, we used GFP-expressing cell count data from VGluT2-IRES::Cre mice that were injected in the VTA with AAVs encoding Cre-dependent TVA-mCherry and Cre-dependent optimized rabies glycoprotein (OG). Three weeks following the initial helper virus infusion, the mice underwent stereotaxic surgery again and were injected in the VTA with EnvA G-deleted rabies GFP. The image that is being used for demonstration is the same one that is included in the SHARCQ GitHub repository for practice and is an example of retrograde tracing from VGluT2-expressing VTA neurons (Fig. 4*A*). Contrast for the GFP image layer was adjusted to optimize the appearance of labeled neurons, a feature that can be useful for highlighting labeled cells or making brain landmarks such as axon tracts more clear (Fig. 4*B*). When the user selects the “Register Sections to Atlas” action button in the second step, two pop-up windows will appear titled “Slice Viewer” (Fig. 4*C*) and “Atlas Viewer” (Fig. 4*D*). Before transformation, the user must scroll to the correct AP coordinate and DV/ML tilt in the Allen Brain Atlas. For the image used, it was visually determined that the best corresponding coordinate was +1.35 AP. Within both windows, the user must then press the “t” key to trigger the transform mode that will allow the user to click numbered and matching points between the “Slice Viewer” and “Atlas Viewer.” After hitting the “h” key for atlas registration, the “Atlas Viewer” window will be updated with the brain slice overlayed. If the user is not satisfied with the registration, registration points can be added or deleted until the transformed histology matches the atlas, after which the transformation must be saved again. The final image-ROI-atlas composite is created when the user is prompted in the fourth step to “Analyze Section with Allen Atlas” (Fig. 4*E*) or “Analyze Section with Chon Atlas” (Fig. 4*F*). An additional visualization output in an optional fifth step is a 3D rendering of all cell counts from images that have been processed (Fig. 4*G*). In the 3D image window, the user may rotate and zoom in on the brain for optimal viewing. A video demonstrating this functionality can be found at https://www.root-lab.org/sharcq.

**Figure 5.**
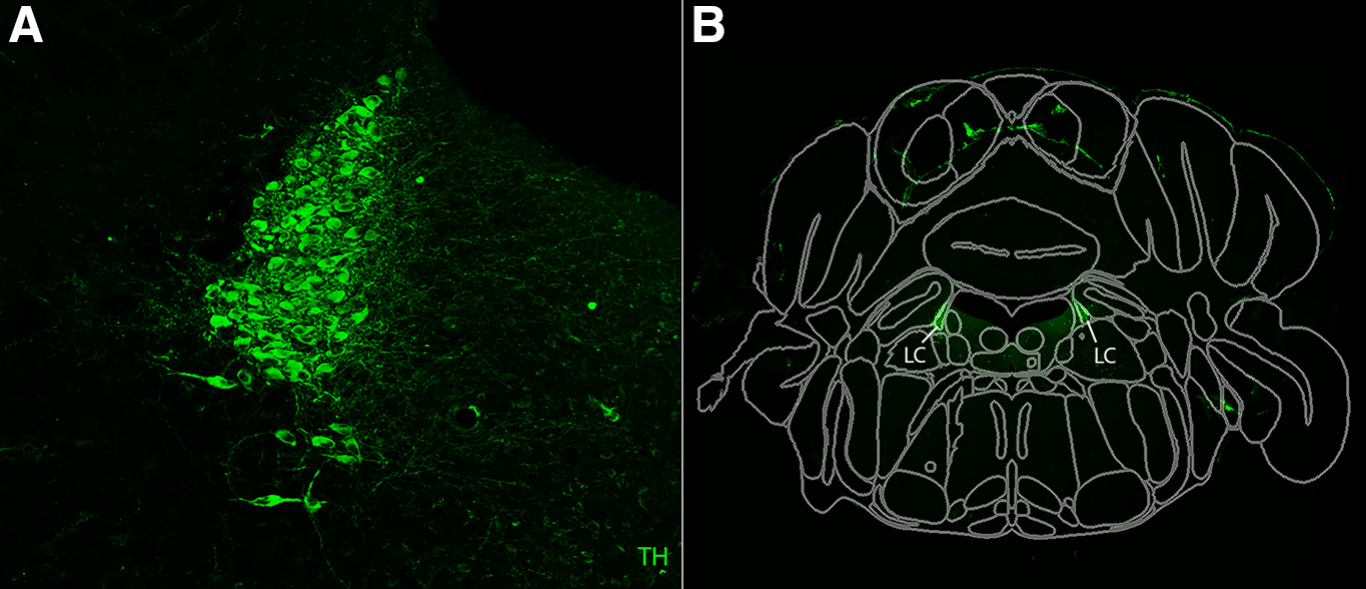
Demonstration of SHARCQ functionality in atlas registration of small brain regions. ***A***, Immunohistochemical labeling of TH-expressing neurons (green) in the LC. Magnification of original slice image used in ***B***. ***B***, Registered slice image-Allen Brain Atlas composite with determined coordinates of −5.34 AP, −1.7° DV tilt, and +3.6° ML tilt. TH-expressing neurons are localized within the borders of the LC.

Additionally, to showcase SHARCQ’s ability to accurately register small brain regions, we immunolabeled TH, a rate-limiting enzyme in dopamine synthesis, in mouse brain slices that contain the locus coeruleus (LC; Fig. 5*A*). SHARCQ processing produced successful registration with TH-positive cells localized within the borders of the LC (Fig. 5*B*).

### SHARCQ functionality is useful across diverse cell labeling techniques

**Figure 6.**
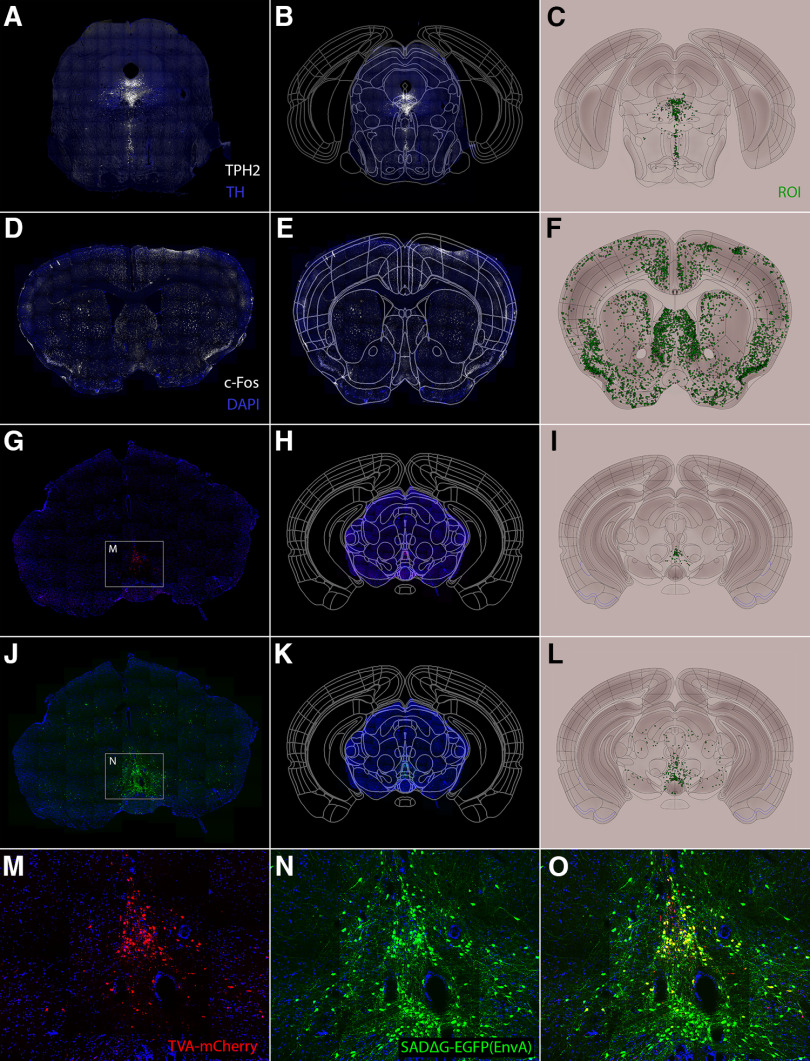
SHARCQ functionality across diverse labeling techniques. ***A***, Histological section with immunofluorescent labeling of TPH2 (white) and TH (blue). ***B***, Registration of section to the Allen Atlas. ***C***, Visualization of TPH2-labeled cells (green) from the updated Franklin–Paxinos Atlas. ***D–F***, SHARCQ processing of histology with immunofluorescent labeling of c-Fos (white) and DAPI nuclear stain (blue). ***G–I***, SHARCQ processing showing TVA-mCherry (red) and DAPI (blue) labeling with counted cells in the ROI corresponding to mCherry-EGFP overlap cells. ***J–L***, SHARCQ processing on the same section in ***G–I*** showing SADΔG-EGFP(EnvA) (green) and DAPI (blue) labeling. SADΔG-EGFP(EnvA) cells are immediate presynaptic partners to TVA-mCherry cells. ***M***, Inset from ***G***. ***N***, Inset from ***J***. ***O***, Merged mCherry and EGFP fluorescence, showing mCherry-EGFP cells (yellow) are immediately downstream of EGFP cells.

To demonstrate the utility of SHARCQ for common neurohistological methods, we performed different cell labeling and counting techniques. In one method, sections containing the dorsal and median raphe were immunolabeled for tryptophan hydroxylase 2 (TPH2; Fig. 6*A*), a rate-limiting enzyme for the biosynthesis of serotonin. The image was then registered to the Allen Atlas using SHARCQ (Fig. 6*B*). The TPH2+ cells were manually counted in FIJI, and the ROIs were registered using the warping function from the image and analyzed using the modified Franklin–Paxinos Atlas (Fig. 6*C*). This demonstrates SHARCQ’s usefulness in whole-brain sampling of cell types of interest. In another method (Fig. 6*D–F*), mouse brain sections were immunolabeled for c-Fos, an immediate early gene indicator, and processed through SHARCQ. This method is particularly useful for unbiased identification of brain regions activated by experimentally-defined events. Further, it demonstrates SHARCQ’s capacity to handle a large density of cells (>5000 c-Fos-expressing neurons within the whole image in this example). In the third method (Fig. 6*G–O*), two separate groups of neurons were manually counted in Photoshop: the neurons co-expressing mCherry and EGFP (Fig. 6*G–I*) and neurons expressing EGFP without mCherry (Fig. 6*J–L*). After processing the mCherry-EGFP cell counts through SHARCQ, the same warping function was applied to the EGFP-only cell counts without the user needing to perform the registration a second time. This application is ideal for analyzing histology with multiple cell populations of interest because it applies identical registration and analysis across cell groups, thereby increasing throughput capacity and eliminating discrepancies in repeated registration.

### Image-ROI-Atlas composition has a high degree of similarity across cell quantification method and atlases

**Figure 7.**
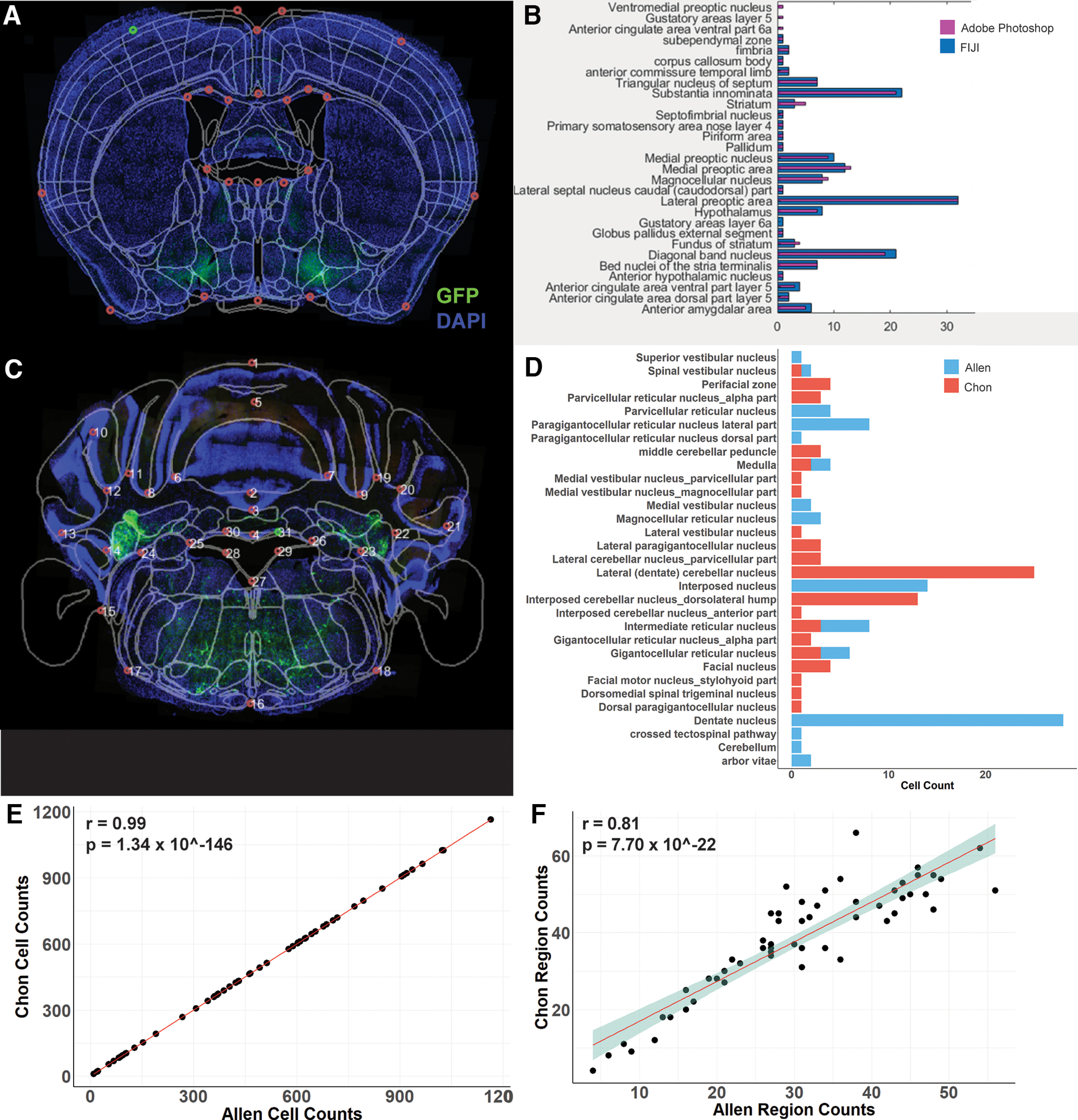
Similar quantification across cell counting method and atlases but discrepancies in brain structure nomenclature between the Allen Atlas and the modified Franklin–Paxinos Atlas. ***A***, Atlas registration example for an image with −0.18 AP coordinate of neurons (green) projecting to VGluT2-expressing VTA neurons with whole cells in DAPI (blue). ***B***, Total cell counts per brain structure of the Allen Atlas between the FIJI counting method and Photoshop counting method. ***C***, Image-to-Atlas registration for an image at −5.94 AP with neurons (green) projecting to VGluT2-expressing VTA neurons with whole cells in DAPI (blue). ***D***, Comparison in cell counts between the Allen Atlas quantification and modified Franklin–Paxinos quantification. ***E***, Whole-brain retrograde traced cell counts between the Allen Atlas and the modified Franklin–Paxinos Atlas (*n* = 57 images). ***F***, Whole-brain structure counts between the Allen Atlas and the modified Franklin–Paxinos Atlas (*n* = 57 images).

In order to validate the reliability of SHARCQ features, we compared the cell quantification results by counting method and the provided atlas options. Immunofluorescent imaging data from Cre-dependent retrograde tracing of VGluT2-expressing neurons within the VTA were used. GFP cell bodies of an image (Fig. 7*A*) were manually counted using both Adobe Photoshop and FIJI (Fig. 7*B*). A separate image slice from the same viral strategy (Fig. 7*C*) was used to compare cell count quantification by brain structure according to the Allen Atlas and the modified Franklin–Paxinos Atlas (Fig. 7*D*). Across 35 brain structures, there was overlap in nomenclature of four structures between the Allen Atlas and modified Franklin–Paxinos Atlas in cell count quantification. While cell counts within images retained fidelity across the whole brain, regardless of atlas choice (Fig. 7*E*), there exists discrepancy between atlases in defined brain borders and structure terminology (Fig. 7*F*). For one whole brain, 57 images were analyzed, and a linear regression analysis was performed on total brain region counts from the Allen Atlas versus total brain region counts from the modified Franklin–Paxinos Atlas (*r* = 0.81, *p* = 7.70 × 10^−22^). While there was little to no difference in cell counts (*r* = 0.99, *p* = 1.34 × 10^−146^), there was significant incongruency in the number of brain structures accounted for between atlases (Franklin–Paxinos: 37.8, SD = 14.7; Allen: 30.1, SD = 12.8) with more brain structures defined in the modified Franklin–Paxinos atlas overall (*t*_(56)_ = 9.13, *p* = 1.11 × 10^−12^). Together, this analysis illustrates that the choice of atlas or counting method will be best determined by each experimenter.

## Discussion

Here, we demonstrate the functionality of a semi-automated workflow and GUI called SHARCQ for registering whole-brain slice images with fluorescently labeled cells onto a whole-brain atlas. The SHARCQ GUI integrates the SHARP-Track toolkit with significant modifications to create a user-friendly interface for registering individual cells of interest to a whole 3D brain atlas. Specific issues were circumvented to achieve this outcome. Namely, during the process of registration, high resolution images were downsampled to match the lower resolution of both the Allen Brain Atlas (Wang et al., 2020) and the Chon Atlas (Chon et al., 2019). With loss of high resolution, cell counting after registration would be imprecise and inaccurate. The SHARCQ GUI requires cell counting before registration, thereby circumventing the issue of down-sampling. As a result, areas of dense cell counts are preserved. The cell counting required before using SHARCQ can be obtained manually or automatically through other means.

MATLAB, a programming platform and language with many capabilities, is proprietary and therefore, not an open-source coding software. The commercial nature of MATLAB can make it difficult for scientists to learn, troubleshoot, and implement MATLAB-based code, especially for scientists with little to no computational background. The SHARCQ GUI was designed to be intuitive, leading a user through every step of the analysis. While options do exist for automated cell counting and atlas registration, it requires extensive code modification or, in the case of machine-learning algorithms, still some level of manual counting for pretraining or computationally intensive training (Fürth et al., 2018; Berg et al., 2019; Tyson et al., 2021). With the SHARCQ GUI, there is no need to interact with or make modifications to the code directly.

The SHARCQ features described here were presented using histology data from various methods, such as Cre-dependent retrograde tracing of VGluT2-expressing neurons within the VTA. SHARCQ was designed with the intention of scientists being able to use it for any type of whole-brain slice histology staining (i.e., HRP-DAB method, fluorescent histochemical methods, autoradiographic staining methods). The only image requirement of SHARCQ is for the image to be a .tif/.tiff file type, which is the most common image file type because of its ability to retain image quality.

A useful feature is the option to choose between the Allen Mouse Brain Atlas or the modified Franklin–Paxinos Atlas for cell count quantification by brain structure (Chon et al., 2019; Wang et al., 2020). The cell counting step exists outside of the SHARCQ interface, and therefore, quantification of cell counts is retained, regardless of the selected atlas. Understanding that there are discrepancies of brain region borders and even nomenclature between the Allen Mouse Brain Atlas and the Franklin–Paxinos Atlas, SHARCQ allows both atlases for cell count quantification. In addition, when quantifying cell counts by atlas, the user has the option to move individual or grouped counted cell ROIs after warping. This feature allows for flexibility in adjusting the coordinates of counted cell ROIs after atlas registration for maximum precision and accuracy in the final cell counts by brain regions.

With regard to data sharing, substantial effort was made to be as transparent as possible with the development of SHARCQ, as well as with all code contributing to its functionality, including steps outside the SHARCQ GUI, such as sample preparation, image acquisition and cell counting. The MATLAB GUI was selected for the development of SHARCQ because of the easy nature of uploading files and point-and-click action buttons. SHARCQ is a conceptually simple framework of an image analysis pipeline. It not only reduces user input to what is only necessary, but in the command window of MATLAB are key stroke directions for navigation and manipulation. While html-based platforms, like RMarkdown or Jupyter Notebook, contribute to data reproducibility with recorded and reproducible code, it was not necessary for the purposes of SHARCQ. Rather, SHARCQ maintains data reproducibility by retaining the original image file and outputs the alterations that were made at every step of the pipeline, including contrast adjustments, down-sampling, and atlas registration.

The rapid development in digitized atlases and atlas registration of images has changed the onerous task of postimaging analysis into a quick, automated output. Instead of drawing borders by hand onto images, researchers can use a GUI such as SHARCQ to automatically determine the number of precounted cell bodies within discrete brain regions. Other programs exist that integrate both automated cell counting and atlas registration (Kopec et al., 2011; Fürth et al., 2018; Pallast et al., 2019; Bourgeois et al., 2021). Notably, some of these programs automate image warping by setting an intensity threshold or conducting a principal component analysis to detect tissue edges, but such an approach is less conducible with irregular tissue, such as a missing hemisphere or torn tissue edge (Kopec et al., 2011; Fürth et al., 2018). The advantage of landmark-based registration, which is an integral feature of SHARCQ, is that regardless of tissue quality, image to atlas registration will occur with high precision based on user-defined landmarks.

In addition, many of these programs require moderate programming experience to achieve the goal of image to atlas registration. Some examples include line modifications to the code script, execution of additional code within the command window, or experience with multiple programs in the pipeline (Kopec et al., 2011; Fürth et al., 2018; Pallast et al., 2019; Bourgeois et al., 2021). SHARCQ was developed as a user-friendly, self-contained MATLAB interface for atlas and cell count registration that requires no coding and only point-and-click user actions. The SHARCQ GUI was designed for step-by-step registration and analysis with an intuitive layout.

While SHARCQ is a considerable improvement over manual brain border drawing and coding intensive programs, SHARCQ has limitations. We have shown that brain regions, regardless of size, can be accurately registered to the corresponding atlas region. However, differences in ventricle size between the slice image and the brain atlas can lead to difficulties accurately registering ventricles. Disproportionate ventricle size can be because of ventricle expansion during transcardial perfusions at the time of brain retrieval or variation between mouse lines. While expanded ventricles can compress the surrounding brain regions, we have found that careful selection of registration points produces a registration of sufficient quality to accurately assign cell counts to their brain regions. A best practice is to focus registration points on anatomic landmarks within the tissue rather than in or surrounding the ventricles. It is noteworthy that for SHARCQ, the user can manually move cell counts after atlas registration as a mechanism to verify and review that cell counts exist in the appropriate brain regions.

A second limitation is that SHARCQ has no integrated automated cell counting feature in its current form. It is worth noting that one of the SHARCQ requirements is an X,Y coordinate file of counts that match the dimensions of the image. Therefore, although it is not demonstrated here, it is possible to use another automated cell counting program that can output the described X,Y coordinate file and then use SHARCQ to register the image and counted cells to an atlas. Automated cell counting by establishing an intensity threshold and binarizing the image can be accomplished in FIJI or MATLAB. Automated cell counting was not integrated into SHARCQ because the process still requires significant user input to optimize thresholding and binarization. The intensity threshold can vary within and between images, establishing an intensity threshold would have to be manually determined by the user. For example, in our 16-bit .tiff image files, in which maximum intensity was 65,535, the manually counted cells did not always have an intensity value of 65,535, and the minimum intensity was not uniform across images.

A third limitation is that neuroscientists who use non-mouse species for research, such as rat, marmoset, or zebrafish, will not find utility in SHARCQ. Recently, the integration of multiple taxa brain atlases, including mouse, human, and larval zebrafish, was achieved by establishing an application programming interface (API) called BrainGlobe (Claudi et al., 2020). BrainGlobe was used to develop Brainrender, a Python-based tool that can integrate visualization of cell counts (Claudi et al., 2021). While Brainrender can produce high quality visualizations, its main limitation is that it is currently not a quantification tool. SHARCQ, while limited to the mouse brain, produces both 2D and 3D visualization of cell counts and also enabling quantification of cell counts by brain regions. With more open-source data sharing and development, SHARCQ can be substantially expanded to different species. For now, SHARCQ satisfies a need for quick, automated cell count quantification across whole mouse brains, made possible through the expansion of currently existing toolkits (e.g., SHARP-Track).

## Synthesis

Reviewing Editor: Mark Laubach, American University Decisions are customarily a result of the Reviewing Editor and the peer reviewers coming together and discussing their recommendations until a consensus is reached. When revisions are invited, a fact-based synthesis statement explaining their decision and outlining what is needed to prepare a revision will be listed below. The following reviewer(s) agreed to reveal their identity: Sean Bradley, Chandrashekhar Borkar.

Two reviewers read your manuscript and provided critical feedback. We also discussed your paper in the discussion forum, and additional concerns were raised there. Please address all points that were raised in your revised manuscript.

Reviewer 1

The authors provide a novel tool dubbed SHARCQ for conducting neurohistological studies of sectioned mouse brain. This package leverages MATLAB’s Image Processing Toolbox and extant digitized brain atlas data to a) assist users in correctly identifying locations in sample sections, b) warp images of an even imperfect sample sections to match those of landmark-matched canonical section from one of two brain atlases, and c) visualize and analyze results in several useful ways.

This software would be useful to those conducting neurohistology, especially those that have troublesome/irregular sections, are conducting tract tracing, or are adopting a whole-brain approach. It is particularly noteworthy for facilitating the use of even poorly mounted sections for mapping/tracing and its high level of accessibility that requires no programming experience.

During my review, I downloaded the sample files (which the authors may consider moving into the distro; they’re a bit hidden under the “Requirements” heading of the wiki) and was able to run through the entire workflow. I found the experience to be largely straightforward and self-explanatory, for which the authors are to be commended; I was able to complete the workflow within some 30 minutes of download which is remarkable for a rather complex endeavor.

I did encounter several errors that were caused by my inexperience. Improved error messages might be a worthwhile addition in the future, but aren’t a requested revision.

Apparent bug:

Selecting “Analyze with Allen Brain Atlas” prompts for .csv instead of .tif.

Compatibility issue:

Although the documentation indicates that the software should work with “2020b or more recent,” the software did not seem to work with MATLAB 2021b. The ROI .csv seems to be read incorrectly. The authors may wish to amend their stated compatibility or address the issue.

Regarding Figure 6, Panel E: I’m unclear regarding what this figure means to demonstrate. The figure depicts an identical relationship between the whole-slice cell counts of some 57 images when analyzed via the two atlases. This seems like a somewhat tautological idea; the information provided to both analyses (via the counts from the ROI .csv files) is the same, so of course they should be identical. How would loss of counts be introduced by a change in atlas? What is being ruled out? Or are those data from within structures that share nomenclature?

Reviewer 2

The authors in the manuscript entitled “A semi-automated workflow for brain Slice Histology Alignment, Registration, and Cell Quantification (SHARCQ)” describe the post-imaging analysis tool called SHARCQ (Slice Histology Alignment, Registration, and Cell Quantification) to map histological images in the established brain atlases. This is a novel, efficient and interesting tool, allowing users to perform cell-to-brain registration and quantification with a simple, accessible interface by using Matlab software. Authors have provided all the codes to download and use freely. This semi-automated tool has several advantages including accurately registering brain area and counting histology data. In addition, it allows friendly interface for registering individual cells of interest to a whole 3D brain atlas. The main disadvantage of technique is that it requires paid/commercial software to use it and cell counting needs to be performed using other software. However, this technique may be helpful to wide variety of scientists in the field, thus worth accepting for publication after addressing few concerns.

Comments:

1. Authors enlist limitation of this tool as it is useful in only in mouse, although rat brain atlases are available. Authors need to clarify if this tool can also be upgraded for rat brain.

2. Authors need to add more instructions on slice mounting procedure as the change in the orientation or improper sections mounting could lead to incorrect area selection as depicted in left cortical part of figure 6A.

3. Authors need to add more clarification on how the brain sections are being allotted to their respective anterio-posterior coordinate coronal sections from the atlas.

4. Do authors recommend the thickness of the sections for correct registration of the brain areas in atlas?

5. Authors need to show how correct registration of the brain areas works for the cell counting feature in the smaller areas like claustrum or locus coeruleus. The counting also looks inconsistent in figure 6D. Is that because of the difference in area registration?

Feedback from the discussion forum:

- Please address all points raised by both reviewers. Please check on reported usage bugs and Matlab version compatibility.

- Please respond and revise to address the point made by Reviewer 1 at the end of the review (on Figure 6E).

- Please revise to address all points made by Reviewer 2. Some additional comments are below for the points that Reviewer 2 raised.

1. Authors address the current inability of this software to be used with non-mouse species and their future intent to permit analysis on a wider variety of taxa on merged doc p17. The manuscript should be revised to emphasize species limitations as early as possible in the text (Introduction).

2. With respect to Figure 6A, the small extrusion of cortex from the fitted model could conceivably be corrected with additional matched points for the warp (or might be a product of histological prep / natural variation). A detailed explanation of slide prep might be beyond the scope of the paper (and a citation of a protocol paper might be nice), an explanation of a) any expectations/assumptions for slide prep made by the software and b) how many matched points to use and how to choose them (especially for suboptimal slices) would be useful to potential users. Please address these issues in the revised submission.

- Demonstrating validity would be nice (can you get the same results from adjacent slices with an axonal reporter?) but because the registration/warping is ultimately in users-controlled, giving additional guidance on best practices for good results would be useful here.

- Four recent open source tools were cited: Shamash et al., 2018; Pallast et al., 2019; Bourgeois et al., 2021; Claudi et al., 2021. These tools are mentioned early in the manuscript and in the last paragraph of the discussion. More extensive discussion on how the new tool compares to these recent ones is warranted. What does the new tool provide that these tools do not?

- brainrender from Claudi and colleagues is becoming an increasingly used tool, with general utility. The authors should include extensive statements about how their tool compares to it and the others.

## Author Response

We thank the reviewers and editor for their valuable feedback on the initial submission. We have updated the manuscript according to their feedback and believe that manuscript is greatly improved. Our responses to initial feedback are documented in blue within this letter and within the revised manuscript.

Original Review Below:

Significance Statement Comments for Author (Required):

N/A

Comments on the Visual Abstract for Author (Required):

Please consider providing a visual abstract for this paper. I might have missed it but did not see it in the submission files. This helps with promotion of your tool.

We have submitted a visual abstract in the revised manuscript.

Synthesis Statement for Author (Required):

Two reviewers read your manuscript and provided critical feedback. We also discussed your paper in the discussion forum, and additional concerns were raised there. Please address all points that were raised in your revised manuscript.

Reviewer 1

The authors provide a novel tool dubbed SHARCQ for conducting neurohistological studies of sectioned mouse brain. This package leverages MATLAB’s Image Processing Toolbox and extant digitized brain atlas data to a) assist users in correctly identifying locations in sample sections, b) warp images of an even imperfect sample sections to match those of landmark-matched canonical section from one of two brain atlases, and c) visualize and analyze results in several useful ways.

This software would be useful to those conducting neurohistology, especially those that have troublesome/irregular sections, are conducting tract tracing, or are adopting a whole-brain approach. It is particularly noteworthy for facilitating the use of even poorly mounted sections for mapping/tracing and its high level of accessibility that requires no programming experience.

During my review, I downloaded the sample files (which the authors may consider moving into the distro; they’re a bit hidden under the “Requirements” heading of the wiki) and was able to run through the entire workflow. I found the experience to be largely straightforward and self-explanatory, for which the authors are to be commended; I was able to complete the workflow within some 30 minutes of download which is remarkable for a rather complex endeavor.

I did encounter several errors that were caused by my inexperience. Improved error messages might be a worthwhile addition in the future, but aren’t a requested revision.

We were unable to alter error messages as integrated within MATLAB. However, as an alternative we have updated the troubleshooting page on the SHARCQ Github repository to address major issues that may be encountered.

Apparent bug:

Selecting “Analyze with Allen Brain Atlas” prompts for .csv instead of .tif.

This feature now correctly prompts for a .tif file and not a .csv file in the new popup window.

Compatibility issue:

Although the documentation indicates that the software should work with “2020b or more recent,” the software did not seem to work with MATLAB 2021b. The ROI .csv seems to be read incorrectly. The authors may wish to amend their stated compatibility or address the issue.

We have checked compatibility with R2021b and encountered no issues running the SHARCQ pipeline. In the manuscript, we now specify that either 2020b or later MATLAB installation is required. We suspect that some of the issues during review may have been caused by a lack of the Image Processing Toolbox in each MATLAB version and we have also specified this requirement for successful use.

Regarding Figure 6, Panel E: I’m unclear regarding what this figure means to demonstrate. The figure depicts an identical relationship between the whole-slice cell counts of some 57 images when analyzed via the two atlases. This seems like a somewhat tautological idea; the information provided to both analyses (via the counts from the ROI .csv files) is the same, so of course they should be identical. How would loss of counts be introduced by a change in atlas? What is being ruled out? Or are those data from within structures that share nomenclature?

We have attempted to make the wording describing this figure panel more clear. Ultimately, the purpose of Figure 6E was to emphasize the discrepancy in Figure 6F. While cell counts are retained regardless of selected brain atlas, there will be an incongruency in allotted cell counts to brain structures. This is because there are more brain structures defined in the modified Franklin-Paxinos atlas than the Allen atlas, and in the rare case, vice versa. This point is clarified by adding the results from a paired samples t-test comparing the counted brain structures between atlases in the Results subsection “Image-ROI-Atlas composition has high degree of similarity across cell quantification method and atlases”.

Reviewer 2

The authors in the manuscript entitled “A semi-automated workflow for brain Slice Histology Alignment, Registration, and Cell Quantification (SHARCQ)” describe the post-imaging analysis tool called SHARCQ (Slice Histology Alignment, Registration, and Cell Quantification) to map histological images in the established brain atlases. This is a novel, efficient and interesting tool, allowing users to perform cell-to-brain registration and quantification with a simple, accessible interface by using Matlab software. Authors have provided all the codes to download and use freely. This semi-automated tool has several advantages including accurately registering brain area and counting histology data. In addition, it allows friendly interface for registering individual cells of interest to a whole 3D brain atlas. The main disadvantage of technique is that it requires paid/commercial software to use it and cell counting needs to be performed using other software. However, this technique may be helpful to wide variety of scientists in the field, thus worth accepting for publication after addressing few concerns.

Comments:

1. Authors enlist limitation of this tool as it is useful in only in mouse, although rat brain atlases are available. Authors need to clarify if this tool can also be upgraded for rat brain.

We have updated the last paragraph of the Introduction to clarify that SHARCQ can only be used as a mouse brain histology tool in its current form. In addition, in the last paragraph of the discussion, we also discuss the utility of BrainGlobe, a Python-based API for multiple taxa brain atlases (mouse, human, larval zebrafish with intent of their authors to expand).

2. Authors need to add more instructions on slice mounting procedure as the change in the orientation or improper sections mounting could lead to incorrect area selection as depicted in left cortical part of figure 6A.

Regarding sample preparation, we have added procedural details to optimize imaging and registering samples. For example, we have found that bubbles in mounting medium can significantly obstruct sections under the microscope and subsequent correct registration. We have also added details on sample preparation in the Materials and Methods subsection, “Whole-brain fluorescent labeling of neurons", and included new comments regarding best practices.

3. Authors need to add more clarification on how the brain sections are being allotted to their respective anterio-posterior coordinate coronal sections from the atlas.

In the Materials and Methods subsection “image-atlas registration,” AP coordinate registration is described, which is a manual determination at the user’s discretion. The following sentence was added to the subsection to further clarify this point: “Scrolling to the approximate bregma location is at the discretion of the user, and it is advised that the user has moderate familiarity with neuroanatomy. Registering axon tracts are useful for this purpose (e.g., anterior commissure, corpus collosum, medial lemniscus, etc). “

It is noted again in the Results subsection “A simplified workflow for adapting multiple software for cell count quantification to atlas registration” in the third paragraph: “In the ’Atlas Viewer’ window, the user must scroll to the approximate location in the brain that matches the slice in the ’Slice Viewer’ window. It is at the discretion of the user to determine the correct coordinate and tilt, if necessary.”

4. Do authors recommend the thickness of the sections for correct registration of the brain areas in atlas?

We have only used 30μm sections for our purposes, which is noted in the Materials and Methods subsection “Whole-brain fluorescent labeling of neurons.” Each lab/user may have their own protocol for histology in order to maximize image quality. We have found that high image resolution was best for our manual cell counting protocol.

5. Authors need to show how correct registration of the brain areas works for the cell counting feature in the smaller areas like claustrum or locus coeruleus. The counting also looks inconsistent in figure 6D. Is that because of the difference in area registration?

We have included a new figure registering bilateral locus coeruleus within SHARCQ and a discussion on the strengths and weaknesses of our approach. The inconsistency in Fig 6D is explained by Fig 6F, which highlights the discrepancy in the number of brain regions defined by the Franklin-Paxinos atlas and the Allen atlas. The results from a paired samples t-test comparing the counted brain structures between atlases was added to the Results subsection “Image-ROI-Atlas composition has high degree of similarity across cell quantification method and atlases”.

Feedback from the discussion forum:

- Please address all points raised by both reviewers. Please check on reported usage bugs and Matlab version compatibility.

We have been unable to recreate most of the reported bugs. In our tests we were able to run the entire SHARCQ pipeline on 2021b. We suspect that the main issue was a lack of the Image Processing Toolbox for the recently downloaded MATLAB version. Nevertheless, we have updated the troubleshooting page of the SHARCQ Github repository to address potential issues.

- Please respond and revise to address the point made by Reviewer 1 at the end of the review (on Figure 6E).

We have included a new figure registering bilateral locus coeruleus within SHARCQ and a discussion on the strengths and weaknesses of our approach (e.g., differences in ventricle sizes between atlas and image that can be problematic). The inconsistency in Fig 6D is explained by Fig 6F, which highlights the discrepancy in the number of brain regions defined by the Franklin-Paxinos atlas and the Allen atlas. The results from a paired samples t-test comparing the counted brain structures between atlases was added to the Results subsection “Image-ROI-Atlas composition has high degree of similarity across cell quantification method and atlases”.

- Please revise to address all points made by Reviewer 2. Some additional comments are below for the points that Reviewer 2 raised.

1. Authors address the current inability of this software to be used with non-mouse species and their future intent to permit analysis on a wider variety of taxa on merged doc p17. The manuscript should be revised to emphasize species limitations as early as possible in the text (Introduction).

In both the Introduction and Discussion we have clarified that SHARCQ can only be used as a mouse brain histology tool in its current form.

2. With respect to Figure 6A, the small extrusion of cortex from the fitted model could conceivably be corrected with additional matched points for the warp (or might be a product of histological prep / natural variation). A detailed explanation of slide prep might be beyond the scope of the paper (and a citation of a protocol paper might be nice), an explanation of a) any expectations/assumptions for slide prep made by the software and b) how many matched points to use and how to choose them (especially for suboptimal slices) would be useful to potential users. Please address these issues in the revised submission.

Regarding sample preparation, we added detail to minimize the appearance of bubbles on slides as much as possible. We have found that bubbles significantly obstructed sections under the microscope leading to poor registration. Additional details of optimal sample preparation have been included in Materials and Methods subsection, “Whole-brain fluorescent labeling of neurons”. It is highly advised that at least 10 landmark points are clicked and matching between the ‘Atlas Viewer’ and ‘Slice Viewer’ windows. This is noted in the Materials and Methods section and again in the Results. See below for our comments regarding best practices as well.

- Demonstrating validity would be nice (can you get the same results from adjacent slices with an axonal reporter?) but because the registration/warping is ultimately in users-controlled, giving additional guidance on best practices for good results would be useful here.

We have made the best practices more explicit, including:

• Minimum of 10 landmark points that match between the ‘Atlas Viewer’ and ‘Slider Viewer’ windows

• Adjust contrast in the “pre-processing image” step to better discern landmarks

• Same size, aspect ratio, and orientation is required between the X,Y coordinate file and the image file

• Original image and X,Y coordinate file must be in the correct anatomical orientation, meaning if it is a coronal section, then dorsal is “up” and ventral is “down.”

• Background channel for cell labeling, like DAPI or another cell marker

• In the “pre-processing image” step, the user must click through all images, if there are multiple channels, using the arrow keys for review. This step converts the image channels to the same aspect ratio as the Allen atlas for image-atlas registration.

- Four recent open source tools were cited: Shamash et al., 2018; Pallast et al., 2019; Bourgeois et al., 2021; Claudi et al., 2021. These tools are mentioned early in the manuscript and in the last paragraph of the discussion. More extensive discussion on how the new tool compares to these recent ones is warranted. What does the new tool provide that these tools do not?

Kopec et al. 2011 was added as another tool that incorporated cell counting with atlas registration. The discussion was revised to include an expanded commentary on the differences and improvements made with SHARCQ. Claudi et al., 2021 was moved to a different paragraph to separately discuss and compare its utility to SHARCQ.

- brainrender from Claudi and colleagues is becoming an increasingly used tool, with general utility. The authors should include extensive statements about how their tool compares to it and the others.

Brainrender (and BrainGlobe) was added to the last paragraph of the discussion to highlight that its main utility is the incorporation of multiple taxa atlases, primarily mouse, human, and larval zebrafish (adding other taxa is still under development, according to their authors). However, we note that the primary limitation of Brainrender, while it is a highly useful and impressive visualization tool, is that it is not a cell counting quantification tool currently. SHARCQ is a 2D,3D visualization tool, as well as a quantification tool.
